# Procrastination and Personal Finances: Exploring the Roles of Planning and Financial Self-Efficacy

**DOI:** 10.3389/fpsyg.2019.00775

**Published:** 2019-04-05

**Authors:** Thor Gamst-Klaussen, Piers Steel, Frode Svartdal

**Affiliations:** ^1^Department of Psychology, UIT The Arctic University of Norway, Tromsø, Norway; ^2^Organizational Behaviour and Human Resources, University of Calgary, Calgary, AB, Canada

**Keywords:** procrastination, planning, financial behavior, financial self-efficacy, impulsivity

## Abstract

Procrastination is related to unhealthy personal financial behaviors, such as postponing retirement savings, last minute shopping, and not paying bills on time. The present paper explores factors that could explain why procrastinators demonstrate more financial problems compared to non-procrastinators. Study 1 (*N* = 675) focused on planning, as both procrastination and poor financial habits are negatively related to planning. Results confirmed that procrastination was a significant predictor of personal finances, but the propensity to plan was not. Study 2 (*N* = 500) explored the roles of procrastination and financial self-efficacy in two facets of financial behavior, financial impulsivity and financial planning. Results indicated that the effect of procrastination on financial behavior was fully mediated by financial self-efficacy. Hence, these results suggest that procrastination operates primarily through its self-efficacy component to impact financial behavior negatively.

## Introduction

Financial behaviors and decisions related to personal investments, mortgages, retirement, and savings require quite complex knowledge and skills, and healthy financial decisions require an ability to recognize long-term consequences of current choices (Brown and Poterba, 2006; Lusardi and Mitchell, 2007, 2011; Nye and Hillyard, 2013). Unfortunately, people’s decisions related to personal finance are often suboptimal. Credit card debt has become a major economic problem, increasing to an all-time high alongside household debt (FRB, 2018; TMC, 2019). Delaying payment on credit card debt incurs a high interest rate, and postponement of paying bills is a common way of handling payment problems (Keys and Wang, 2019). As a result, debt collection agencies get increasingly more cases (Fedaseyeu, 2015). A report from 2011 indicated that 87% of United States students had a credit card debt with an average balance of $800 (Nye and Hillyard, 2013). Studies also show that student credit card debt and student loans are increasing, especially in countries with expensive tuition fees (Crawford and Jin, 2014; Feiveson et al., 2018). As healthy personal finances are positively related to psychological health and well-being (Mills et al., 1992; Kim et al., 2003; Richardson et al., 2017; Oskrochi et al., 2018) and higher productivity (Kim and Garman, 2003), an improved understanding of factors related to personal financial behaviors and decisions is important also from a health perspective.

While lack of financial knowledge can compromise financial behavior, a lack of rationality itself can potentially contribute as well. Many of the detrimental consequences of poor personal finances are linked to impulsivity and reduced appreciation of the long-term consequences of current choices, suggesting that at least some unhealthy financial behaviors are an example of procrastination (Steel, 2007, 2010). Procrastination, the voluntary delay of an intended course of action *despite expecting to be worse off due to the delay* (Steel, 2007), is strongly associated with impulsivity and present-bias preferences (Steel et al., 2018). One form of a present-bias preference is that the individual impulsively diverts from a planned course of action, turning to something more pleasurable instead. Such diversions are themselves not delays, but they delay planned behavior indirectly. Another form of the preference is seen when aversive or boring tasks are postponed because performing the same task tomorrow subjectively seems more attractive as near-term costs outweigh distant aversive consequences (Akerlof, 1991; O’Donoghue and Rabin, 1999). Although these delays may be associated with short-term benefits, they are knowingly outweighed by the long-term costs on performance, health, and well-being, subjective as well as financial (Steel, 2007; Steel and Ferrari, 2013).

Studies suggest that 15–20% in the general population chronically procrastinate (Harriott and Ferrari, 1996), with similar estimates found across Australia, United Kingdom, United States, Spain, Peru, and Venezuela (Ferrari et al., 2005, 2007). In the student population, the prevalence of procrastination is double or even triple that of the general population (Steel, 2007; Rozental and Carlbring, 2013), with almost 50% found to procrastinate consistently and problematically (Onwuegbuzie, 2000; O’Brien, 2002). Given this prevalence, it is not surprising procrastination and impulsiveness has been linked to unhealthy financial behavior, such as ballooning card debt (Nye and Hillyard, 2013). For example, Barboza (2018) argued that consuming gives an immediate satisfaction of needs whereas saving is an immediate cost with potential gains in the future. He also suggested that lack of patience and present-bias preference are key determinants for procrastinating credit card debt, resulting in payment of the smallest amount, allowing further consumption. More specifically, individuals with low self-control and those using credit cards to handle unforeseen expenses are more likely to carry credit card debt after the purchase period (Mansfield et al., 2003; Barboza, 2018), as well as more likely to choose costly quick-access credit items such as store cards and payday loans (Gathergood, 2012). Indeed, Meier and Sprenger (2010) found present-biased individuals to be more likely to have credit card debt, as well as significantly higher amounts of credit card debt. Furthermore, present-biased preference explains tax filing close to the deadline, which leads to last-minute mistakes and overpayments (Kasper, 2004; Martinez et al., 2017). Lastly, one study suggested that procrastination is the outcome of present-biased preferences by demonstrating that procrastinators behave differently from non-procrastinators concerning important financial behaviors related to retirement planning, being less likely to participate in saving plans, initiate saving later, and less likely to save a fixed sum every month (Brown and Previtero, 2014).

## Study 1

Detrimental financial behavior becomes procrastination when people voluntarily delay planning or implementing finance-related plans, despite expecting to be worse off for this delay. As reviewed by Steel et al. (2018), impulsive individuals are often poor or absent planners, and a recent study by Steel et al. (2018) demonstrated that planning correlates moderately and negatively with procrastination, *r* = -0.51. Aside from being a symptom of procrastination, a lack of planning can also be a cause. While planning does not necessarily eliminate procrastination, as per the intention-action gap, when it overlaps with motivational techniques like goal setting, it reduces irrational delays. Linking to financial behavior, a recent study found that self-control and deliberate thinking, (i.e., make plans and analyze problems) were important predictors of financial behavior (Strömbäck et al., 2017). Furthermore, Svartdal et al. (2018) demonstrated that procrastinators tend to opt for more expensive lunch habits (buying food in the cantina rather than bringing lunch from home), and suggested poor planning capabilities to be a likely explanation (procrastinators fail to think ahead and prepare lunch package before leaving home). This study further demonstrated that these lunch habits were likely even for participants with lower incomes, suggesting that being in a financially negative position is not enough to motivate the individual to change.

Hence, Study 1 focuses on the relation between planning and procrastination in explaining financially harmful habits. We administered an internet-based survey measuring general planning habits, financial behavior, as well as procrastination.

Hypothesis 1: Planning will predict better financial behavior when controlling for procrastination.

### Methods

#### Participants

As seen in Table 1, 675 adults participated between the ages of 18 to 70 with a mean age of 33.10 (*SD* = 10.59). The majority were female (70.6%) and most had 3 years or more at university (70.5%).

**Table 1:** Sample characteristics.

*N* = 675	Mean (*SD*)	Min	Max
PFI	3.15 (2.29)	0	8
IPS	2.81 (0.81)	1	5
PPS	2.88 (1.09)	0	5
Age	33.09 (10.52)	18	70
**Gender N (%)**			
Female	476 (70.6)		
Male	198 (29.4)		
**Income N (%)**			
Below 450 NOK	359 (53.3)		
450 NOK or more	315 (46.7)		
**Education N (%)**			
High school or less	199 (29.5)		
University 3-years	170 (25.2)		
University 5-years	306 (45.3)		
**Sector N (%)**			
Student	234 (34.9)		
Public sector	263 (39.3)		
Private sector	124 (18.5)		
Other	49 (7.3)		


#### Procedure and Ethics

The survey included questions about demographics, (i.e., gender, age, income, and education), personal economy and habits, and procrastination, given in that order. Participants were recruited by circulating the survey on social media. All participants were given information about the purpose of the study together with a link to the survey, which was provided through the Qualtrics online survey system^1^. Participants were informed that participation was voluntary and anonymous and that they could withdraw from the study at any time. All gave online informed consent by confirming that they had read and agreed to the information by pressing a “start survey” button. The current project is a part of a larger study on procrastination, which has ethical approval from the Regional Ethical Board in Tromsø, Norway (REK nord 2014/2313).

#### Materials

All participants responded to the Propensity to Plan Scale (PPS) (Lynch et al., 2010) and the Irrational Procrastination Scale (IPS) (Steel, 2010), as well as questions addressing personal finances. The PPS includes six items asking about tendencies to plan for time use in the short run, (e.g., “I set goals for the next few days for what I want to achieve with my time” (Item 1); “I actively consider the steps I need to take to stick to my time schedule the next few days” (Item 3). All items are rated on a 6-point Likert scale, with higher scores indicating more planning. Procrastination was measured using the six-item version of the IPS (Steel, 2010; Svartdal and Steel, 2017), which asks about irrational delay of intended behavior. Items are rated on a 5-point Likert scale, with higher scores indicating more procrastination. Finally, we created a Personal Finance Index (PFI) with four questions that address problems in personal finances. Higher sum scores indicate more problems with personal finances. The four questions were:

(1) “*Have you ever got a reminder to pay an unpaid bill, or that the bill has gone to a collection agency”* (No/Yes).
(2) *Research shows that people fail to pay bills for several reasons. Sometimes it is a question of having no money to pay with there and then, but it also happens that bills are not paid because, for example, you delayed paying: (“I post it into the online bank afterward”) or that you just forgot about the bill (“I put the bill aside and forgot about it”). To what extent can this happen to you?* (Never, sometimes, often).
(3) *Do you sometimes put off paying bills so you end up getting a reminder or the bill is sent to a debt collection agency (even if you had the money to pay)?* (No/Yes).
(4) *If you have forgotten or delayed paying bills so you get a reminder or it is sent to a debt collection agency (even if you had the economy to pay) how many times has this occurred?* (Never, 1–3, 4–6, 7–9, 10, or more).

All scales demonstrated satisfactory internal consistency, IPS (α = 0.92), PPS (α = 0.87), PFI (α = 0.77).

#### Analysis

Ordinary least squares regression model with robust standard errors was employed. The model was specified with PFI as the dependent variable, IPS, and PPS as independent variables. We also included control variables that potentially could be relevant in the model, age, gender, income, education, and employment status (separate analyses demonstrated that conclusions were identical with or without the control variables). The variance inflation factor was below three, which indicated that there were no problems with multicollinearity among predictors. Age had a non-linear relationship with the dependent variable, which was adjusted for by adding Age^2^ to the model. The education variable has four categories indicating the highest completed level of education. Due to few respondents in the category “Secondary school” (*N* = 12), this category was merged with the “High-school” category. Analyses were performed in Stata version 15.1 (StataCorp LP).

### Results and Discussion

Means and standard deviations of the scales used are listed in Table 1. The Pearson correlation coefficient between PFI and IPS was moderate and positive, *r* = 0.34, *p* < 0.001, indicating that increasing financial problems are related to increasing procrastination. The correlation between PFI and PPS was weak and negative (*r* = -0.14, *p* < 0.001), indicating that increasing financial problems was weakly related to fewer tendencies to plan. Finally, the correlation between PPS and IPS was moderate and negative (*r* = -0.25, *p* < 0.001), indicating that less planning was related to more tendency to procrastinate.

A regression model produced an *R*^2^ = 0.25. As seen in Table 2, the standardized regression coefficients reveal that procrastination was a significant predictor of personal finances, β = 0.33, *p* < 0.001, whereas PPS was not, β = -0.02, *p* = 0.452. This result suggests that failing to plan does not independently explain financial problems, but procrastination does. Hence, lack of planning, at least according to the present data, does not separately explain why procrastinators demonstrate more financial problems compared to non-procrastinators.

**Table 2:** Regression results with Personal Finance Index (PFI) as dependent variable (*N* = 640).

	β	Std. Err.	*ρ*
Procrastination (IPS)	0.334	0.109	<0.001
Propensity to plan (PPS)	0.023	0.081	0.553
Female	0.020	0.192	0.599
Income≥450′	0.028	0.258	0.623
**Completed education**			
Bachelor degree	0.036	0.241	0.436
Master degree	0.025	0.254	0.650
**Employment status**			
Work in public sector	0.058	0.275	0.327
Work in private sector	0.061	0.292	0.215
Other	0.092	0.379	0.032
Age	1.718	0.054	<0.001
Age^2^	-1.581	0.001	<0.001


Previous studies have demonstrated that planning correlates positively with healthy financial behavior (Strömbäck et al., 2017; Topa et al., 2018). In the current study this correlation was significant but weak (*r* = -0.14). One reason for this low correlation could be that planning was measured by the PPS, which emphasizes short-term time management and therefore may not capture long-term financial aspects. Lynch et al. (2010) suggested that the tendency to plan might differ depending on domain, (i.e., time vs. money) and temporality (i.e., short vs. long run). Their findings indicated that people plan more for time in the short run than for the long run but for money, short- and long-run planning differ less. In contrast, people that are financially strapped or materialistic plan more for money than time. Accordingly, the scale used for measuring propensity to plan should preferably be specific to financial planning. Still, the PPS used in the current study include items about people’s propensity to actively consult a planner and to actively plan for what they want to achieve with their time, which should be important in explaining financial behavior, (e.g., paying bills on time). Thus, this scale lets us investigate the importance of short-term planning in a more general sense when considering the effect of procrastination on financial behavior. Additionally, similar significant correlations were produced between the propensity to plan for time and money in the short run with both financial planning (*r* = 0.34 and 0.54) and impulse buying (*r* = -0.31 and -0.35), respectively (Lynch et al., 2010). However, a domain-specific measure of financial planning would allow for a more focused investigation of the relationship between procrastination and financial behavior. Similarly, as a custom scale was used to measure financial behavior, use of an established scale would be advantageous.

Consequently, Study 2 used a domain-specific measure focusing on three aspects of financial planning, impulsivity, saving, and budgeting. Also, Study 2 added another factor of interest to the understanding of the relation between procrastination and disadvantageous financial habits, self-efficacy.

## Study 2

Financial deliberations and decisions are often complex, and it is likely that such deliberations and decisions are influenced not only by actual skills and knowledge (Lusardi and Mitchell, 2014) but also by self-assessment of financial skills and knowledge. Self-efficacy is the belief people have about their capabilities to produce levels of performance (Bandura, 1994). Procrastination correlates negatively with self-efficacy, *r* = -0.38 to -0.44 (van Eerde, 2003; Steel, 2007) and is argued to have a causal connection (Steel et al., 2018), exacerbating the effects of impulsiveness. While self-efficacy does reflect ability, it also impacts motivation as those with lower levels are more likely to quit or reduce effort when encountering challenges or obstacles. This can create a self-fulfilling prophecy, as failure to try creates failure itself. Hence, individuals who doubt their capabilities to handle finances are more likely to reduce effort, making them more susceptible to unhealthy financial behaviors like impulse purchases. If procrastinators demonstrate lower financial self-efficacy, this might explain why procrastinators often end up with financially negative outcomes.

There is indeed a connection between self-efficacy and financial behavior. Tokunaga (1993) found that credit abusers had lower self-efficacy and greater anxiety concerning their finances compared to successful credit users. Furthermore, Engelberg (2007) demonstrated that a sense of economic self-efficacy is higher when younger people stick to saving plans and have careful spending behavior. Likewise, those with higher self-efficacy reported less financial stress (Heckman et al., 2014), perceiving themselves running a lower risk of losing money due to interrupted income, unforeseen expenses, and less successful investments, as compared to their counterparts (Engelberg, 2007). They also had a better sense of financial control, and a better economic understanding and a more optimistic view of their future financial situation. Moreover, studies suggest that financial self-efficacy among young adults is important for promotion of achievement-relevant behaviors (Lorie et al., 2001), financial independence (Lee and Mortimer, 2009; Xiao et al., 2014), and healthy financial behaviors (Danes Sharon and Haberman, 2007; Shim et al., 2015; Herawati et al., 2018). In addition, Serido et al. (2016) demonstrated that the effect of parental financial teaching on financial behavior among first-year college student’s was partially mediated by the students own financial self-efficacy. Finally, self-efficacy has been suggested to be an important link between financial knowledge and financial behavior (Shim et al., 2009).

Hence, Study 2 measured three facets of personal finances, financial impulsivity, financial planning, and financial self-efficacy, as well as procrastination. We expected to observe negative relations between procrastination and financial impulsivity/planning. However, given the literature reviewed, we expect financial self-efficacy to be an important factor. While self-efficacy can be a cause of procrastination, it can also be an effect as well as reflect ability. Given procrastination’s strong relationship with impulsiveness, we propose that financial self-efficacy will partially mediate the relationship between procrastination and the tendency to shop impulsively and more fully mediate to plan finances, (e.g., budget, saving).

Hypothesis 2: Self-efficacy will mediate the relationship between procrastination and financial behavior, as measured by (a) impulse control when shopping, and (b) tendency to make budget/save.

### Methods

#### Participants

Participants were 500 individuals between the ages of 16 to 75 with a mean age of 29.43 (*SD* = 11.65), and mostly females (64%). About half of the sample (49.70%) were students, and less than half had a university degree (42.79%).

#### Procedure

The survey comprised three parts, starting with questions about demographics, (i.e., gender, age, income, education), then questions about personal finances, financial behavior and financial self-efficacy, and finally procrastination. All participants were recruited from social media and were given information about the purpose of the study together with a link to the survey, which was provided through the Qualtrics online survey system (see text footnote 2). Respondents were told that participation was voluntary and anonymous, and they were informed about their rights to withdraw from the study at any time. After receiving brief information about the study, all participants gave online informed consent to participate as in Study 1.

#### Materials

All participants responded to four measurement scales. The Executive Personal Finance Scale (EPFS) (Spinella et al., 2007) includes a 6-item subscale asking about financial impulsivity, such as: “*When I go to the store I end up buying things I didn’t set out to buy,’*’ rated on a 5-point Likert scale (1 = never; 5 = always). In the analyses, all items were reversed, higher scores indicating higher impulsive control, (i.e., less tendency for impulsive shopping). This scale will be referred to as Financial Behavior-Impulse Control (FB-IC). The Financial Behavior Scale (FBS) (Nye and Hillyard, 2013) includes a 3-item subscale asking respondents to consider behavioral statements about saving (FBS-S; e.g., *save for important purchases* and *save for unexpected expenses*) and budgeting (FBS-B; e.g., *follow up monthly budget*), rated on a 5-point scale (1 = never; 5 = frequently). Combined, this scale will be referred to as Financial Behavior-Saving/Budgeting (FB-S/B). The Financial Self-Efficacy Scale (FSES) (Lown, 2011) consists of 6 items that describe behavioral aspects of personal financial management, rated on a 4-point Likert scale (1 = exactly true; 4 = not at all true). In the current study, two items deemed less suitable for a student and/or a relatively young population was initially removed before data collection, (i.e., “*It is challenging to make progress toward my financial goals*,” “*I worry about running out of money in retirement”)*. In addition, a third item was subsequently omitted, (i.e., “*When unexpected expenses occur, I usually have to use credit”*), since few Norwegian students reported to use a credit card for such events. Thus, three items measured financial self-efficacy, “*It is hard to stick to my spending plan when unexpected expenses arise*,” “*When faced with a financial challenge, I have a hard time figuring out a solution*,” and “*I lack confidence in my ability to manage my finances*.” Procrastination was measured using the 6-item version of the IPS, which asks about irrational delay of intended behavior rated on a 5-point Likert scale, higher scores indicating more procrastination (Svartdal and Steel, 2017).

#### Model Specification and Estimation

Two models were specified depicting that the influence of procrastination on financial behavior is mediated through financial self-efficacy. The models differ only in terms of the outcome, where financial behavior is indicated by either financial impulsivity (Model 1) or the tendency to save/make a budget (Model 2). Control variables included gender (Male = 0; Female = 1), age categories (16–20 = 0) (21–25 = 1) (26–30 = 2) (31–35 = 3) (36–40 = 4) (41–45 = 5) (46–50 = 6) (51–70 = 7), education (high school or less = 0; university = 1), and income (Norwegian kroner) (0 = less than 300’; 1 = 300’to 600’; 3 = above 600’).

Structural equation models were employed using weighted least squares parameter (WLSMV) estimation, which is appropriate when manifest variables are categorical or ordinal. Model fit to data was examined using standard fit indices, i.e., chi-square test, the comparative fit index (CFI), the Tucker-Lewis index (TLI), root-mean-square error of approximation (RMSEA), weighted root-mean-square residual (WRMR). CFI and TLI values greater than 0.95 indicate good fit (Hu and Bentler, 1999), a WRMR close to 1.00 indicates good fit (Yu, 2002), and RMSEA less than 0.05 indicates close fit (MacCallum et al., 1996). Unstandardized parameter estimates are reported with bias-corrected bootstrap confidence intervals estimated based on 10000 bootstrap draws (MacKinnon et al., 2004). Analyses were performed with Mplus version 8.

### Results and Discussion

**FIGURE 1:**
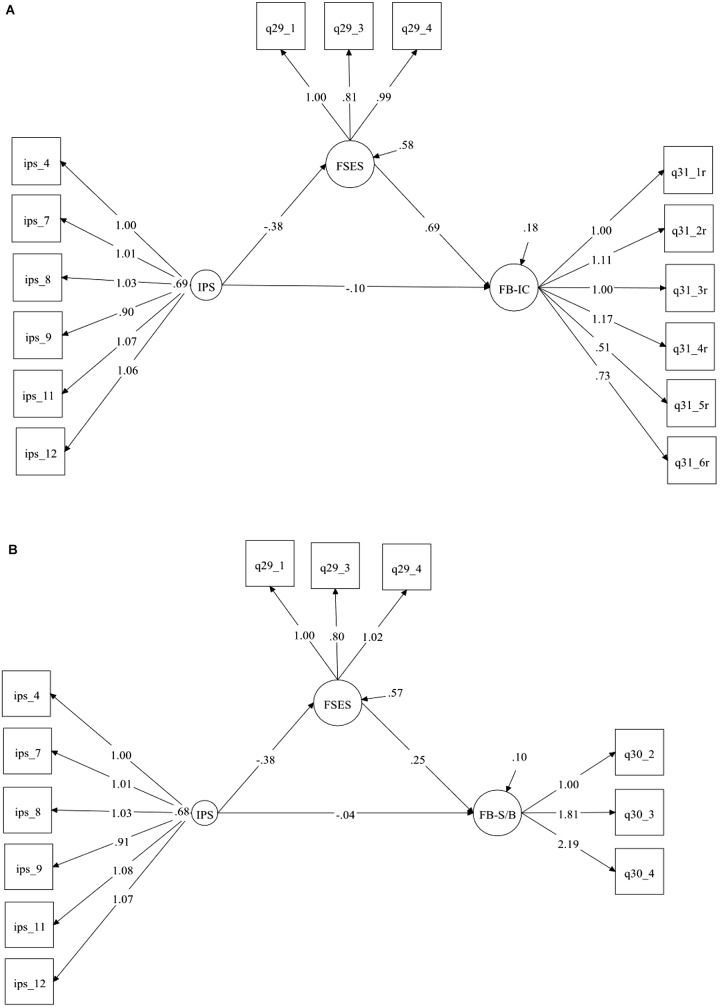
**(A,B)** The estimated conceptual model (unstandardized estimates). See text for explanation. IPS, irrational procrastination scale; FSES, financial self-efficacy scale; FB-IC, financial behavior-impulse control; FB-S/B, financial behavior-saving/budgeting.

All scales indicated a satisfactory internal consistency, IPS (α = 0.92), FSES (α = 0.75), FB-IC (α = 0.80), with the exception of FB-S/B (α = 0.69), which is just below the recommended cut-off criterion (α = 0.70) (Kline, 2015). The correlation coefficients of the main variables indicate that the outcome in both models was significantly (*p* < 0.001) related to the explanatory variables. Descriptive statistics and correlations are displayed in Table 3. The model fit is shown in Table 4. The chi-square test is usually significant with larger sample sizes (Hooper et al., 2008), as was the case in this study. However, several alternative fit indices were examined which suggest that the model fitted the data well. Figure 1 reveals the estimated conceptual model. The direct, indirect and total effects are shown in Table 5.

**Table 3:** Descriptive statistics with Pearson *r.*

	Mean (*SD*)	Min–max	IPS	FSES	FB-IC	FB-SB
IPS	17.26 (5.59)	6–30	1.00			
FSES	9.37 (2.03)	3–12	-0.38^∗^	1.00		
FB-IC	22.01 (4.28)	8–30	-0.38^∗^	0.64^∗^	1.00	
FB-SB	9.07 (3.93)	3–15	-0.30^∗^	0.37^∗^	0.30^∗^	1.00


*Based on simple summation of scale items. IPS, Irrational procrastination scale; FSES, Financial self-efficacy scale; FB-IC, Financial behavior-impulse control; FB-S/B, Financial behavior-Saving/budgeting. ^∗^Pearson *r* sig at <0.001*.

**Table 4:** Model fit indices (*N* = 450).

	*Model 1 (FB-IC)*	*Model 2 (FB-S/B)*
Chi-square	400.620, *df* = 217, *p* < 0.001	280.409, *df* = 151, *p* < 0.001
CFI	0.978	0.983
TLI	0.975	0.979
RMSEA	0.043 (0.037–0.050)	0.044 (0.036–0.052)
WRMR	1.128	1.141


*CFI, comparative fit index; TLI, Tucker-Lewis index; RMSEA, root-mean square error of approximation; WRMR, weighted root-mean-square residual*.

**Table 5:** The direct and indirect effect of procrastination on financial behavior measured as financial impulse control (Model 1) and tendency to save/make budget (Model 2).

	Coefficient (β)	SE	*p*
		**Model 1**	

**Direct effects**			
IPS->FSES	-0.382	0.065	0.000
IPS->FB-IC	-0.096	0.050	0.054
FSES->FB-IC	0.686	0.063	0.000
**Indirect effects**			
IPS via FSES	-0.262	0.048	0.000
Total effect	-0.322		

		**Model 2**	

**Direct effects**			
IPS->FSES	-0.380	0.066	0.000
IPS->FB-S/B	-0.044	0.039	0.262
FSES->FB-S/B	0.248	0.049	0.000
**Indirect effects**			
IPS via FSES	-0.094	0.027	0.000
Total effect	-0.203		


**N* = 450. IPS, Irrational procrastination scale; FSES, Financial self-efficacy scale; FB-IC, Financial behavior-impulse control; FB-S/B, Financial behavior-Saving/budgeting*.

Examination of the direct effects, as they appear in Figure 1, reveals that FSES decreases as a function of procrastination (IPS) (β = -0.382, boot *SE* = 0.07, *p* < 0.001), which was in turn positively related to Financial Behavior as measured by FB-IC (β = 0.686, boot *SE* = 0.063, *p* < 0.001) and FB-S/B (β = 0.248, boot *SE* = 0.049, *p* < 0.001). The direct effect from IPS was non-significant both when FB-IC was the outcome (β = -0.096, boot *SE* = 0.050, *p* = 0.054) and when FB-S/B was the outcome (β = -0.044, boot *SE* = 0.039, *p* = 0.262). In other words, the less procrastination respondents reported, the higher was their financial self-efficacy, which was associated with healthier financial behaviors. This is especially unexpected for financial impulsivity as procrastination is strongly linked to impulsiveness and impulsiveness is a distinct construct from self-efficacy.

This indirect effect of procrastination (IPS) on financial behavior through financial self-efficacy was significant, β = -0.262, boot *SE* = 0.048, 95% Bias-corrected CI [-0.360, -0.186] and β = -0.094, boot *SE* = 0.027, 95% Bias-corrected CI [-0.156, -0.057]. This represents a relative large effect, *k*^2^ = 0.332 and 0.198, respectively. The result of *post hoc* power analysis (Kenny, 2017) given the sample size (*n* = 450), an alpha level of 0.05, and the betas in the model give a power level above 0.80 in all cases, as recommended by Cohen (1988). The only exceptions were the direct effects, which has a power of 0.78 (FB-IC) and 0.15 (FB-S/B). These coefficients are small -0.096, *p* = 0.054 and -0.044, *p* = 0.226. To achieve power of 0.80 for these effects, a sample size of *n* = 474 and *n* = 4404 is needed, respectively.

## General Discussion

The objective of this paper was to investigate factors that could explain why procrastinators report more financial problems compared to non-procrastinators. Study 1 found that procrastinators’ disadvantageous financial behavior was not due to lack of short-term time planning. Study 2 investigated self-efficacy as a mediator of the effect of procrastination on financial behavior, demonstrating that self-efficacy seems to be a crucial factor mediating the procrastination – financial problems relation.

The finding that procrastinators’ financial problems are not due to a lack of planning is consistent with procrastinators’ observed intention-action gap (Steel et al., 2018). That is, procrastinators are just as likely to make intentions to act but have trouble implementing these plans. Still, specific or more advanced forms of planning can play a role. For instance, a procrastinator may consider “when is the latest possible time I must pay my bills” instead of “when is the earliest opportunity to pay my bills.” The former type of planning behavior would make a person more vulnerable to delay or just forgetting to do the task and are probably less likely to take advantage of opportunities to get the task done at an earlier convenience (Lynch et al., 2010). Furthermore, studies have shown that being more specific in the planning stage makes individuals more effective in the implementation of intentions (Wieber and Gollwitzer, 2010). For instance, “I will read that book chapter tomorrow morning at 8 o’clock in the library” vs. “I will read that book chapter tomorrow.” Such planning strategies promoted goal attainment for students at low to moderate levels of conscientiousness but did not affect students at high levels of conscientiousness (Webb et al., 2007). Hence, rather than concluding that planning is not important in financial procrastination and behavior, future studies should examine how planning can be made effective and important. Moreover, planning itself can be delayed, and delayed planning is more common in procrastinators^2^.

Study 2 tested the hypothesis that self-efficacy mediates the relationship between procrastination and financial behavior, as measured by (a) impulse control when shopping and (b) tendency to save/make a budget. The results suggested that the effect of procrastination on financial behavior was completely mediated through financial self-efficacy. Though some mediation was expected, given that self-efficacy is explicitly a causal factor for procrastination (Steel, 2007), what is surprising is the degree. While procrastinating finances would be a subset of unhealthy financial behaviors, financial impulsivity was expected to be more strongly linked to procrastination given the closely related construct of impulsiveness. Still, impulsive spending is a way to enhance self-esteem, improve mood, and reduce stress (Nye and Hillyard, 2013), which connects to self-efficacy as it is negatively related to trait anxiety neurosis, anxiety disorders and depression symptoms (Muris, 2002).

It is clear that self-efficacy is critical to financial health, which is consistent with past mediation research. For instance, self-control and household wealth are positively correlated, and important factors in preventing self-control failure are planning, monitoring, and commitment to pre-set goals (Biljanovska and Palligkinis, 2018), which in turn are all facilitated by people’s confidence in their ability to enact action required to produce the desired outcome (Lown, 2011). Also, procrastination is related to conscientiousness, and a study found that self-efficacy mediated the effect of conscientiousness on subjective happiness (Strobel et al., 2011). Furthermore, personal finances are related to cognitive, (e.g., planning and organization) and emotion factors, (e.g., anxiety and impulsive spending). Individuals with high scores of self-efficacy are optimistic, demonstrate logical thoughts, responsible behaviors, and consistency in affect and mood (Engelberg, 2007; Kiamarsi and Abolghasemi, 2014). Yet another study supported the importance of self-efficacy on interventions targeted at financial behaviors, showing that both the financial knowledge, financial literacy, and self-efficacy are important, but for those who had graduated, financial self-efficacy was the single important factor (Xiao et al., 2014). Overall, knowledge about healthy financial behaviors like saving and budgeting is by itself not enough; people must have the confidence needed to engage in and stay committed to such behaviors.

Of note, these results are of sufficient strength and consistency to direct public policy (Roberts et al., 2007). Given the importance of the financial behavior to the well-being and prosperity of any nation, a key indicator to evaluate the success of any public policy intervention should be an increase in the financial self-efficacy of its people.

## Conclusion, Limitations, and Future Research

One limitation of the present study’s findings, invariably associated with cross-sectional designs, is our difficulty in making definitive causal conclusions (Maxwell and Cole, 2007). Ideally, future studies should try to directly manipulate financial self-efficacy to observe its effects on financial procrastination and behavior. Also, future studies should take into consideration how planning varies in terms of specificity and timing, especially given that procrastinators may benefit from specific and concrete forms of planning that help translating plans into action. Regarding the generalizability of the sample, it was predominantly female, who tend to procrastinate slightly less, though younger, who tend to procrastinate slightly more (Steel and Ferrari, 2013). Though controlling for gender and age did not substantively change the results, using a relatively young population prevents us from effectively assessing larger financial challenges that become more acute later in the lifespan, such as mortgage choices and retirement planning. Given that procrastination is essentially putting off despite expecting to be worse off, we expect that the relationship of procrastination to more serious financial challenges, with their concomitant greater repercussions, (i.e., clearly worse off), the observed relationships should increase. Related to this, it would also be informative to expand the PFI to a broader range of financial behaviors, as it focuses on the eventuality of unpaid bills and associated debts. A more comprehensive assessment could include preceding behaviors such as impulsive purchases, especially those later regretted.

To our knowledge, this is the first study that explores the roles of planning and self-efficacy as mediators on the relationship between procrastination and unhealthy financial behavior. Study 1 found little evidence that lack of short-time planning explains why procrastinators end up with more financial problems. Study 2 demonstrated that self-efficacy completely mediates the association between procrastination and financial behavior. These findings suggest that low self-efficacy may be a key factor to explain why procrastinators suffer financially. However, as discussed, this does not imply that planning is not important for understanding how procrastinators end up with a financial disadvantage, nor that low financial self-efficacy is the only factor responsible for unhealthy financial behavior in procrastinators. Both factors, as well as others, interact over time. For example, since financial self-efficacy reflects perceived ability but it also affects ambition and motivation, it is likely that planning suffers.

## Author’s Note

Data for Study 1 was collected by Eric Elvebø Iversen og Bjørn Anders Karstensen. Ole Christian Sylte and Jesper Solheim Johansen translated the personal finance scales and collected data for Study 2.

## Ethics Statement

The current project is a part of a larger study on procrastination, which has ethical approval from the Regional Ethical Board in Tromsø, Norway (REK nord 2014/2313).

## Author Contributions

TG-K wrote the draft and did the statistical analyses. PS and FS edited the manuscript.

## Conflict of Interest Statement

The authors declare that the research was conducted in the absence of any commercial or financial relationships that could be construed as a potential conflict of interest.

## Footnotes

1 www.qualtrics.com

2 Unpublished data (Svartdal, unpublished) demonstrate that procrastinators tend to plan the same day rather than the day before, whereas non-procrastinators demonstrate an opposite pattern.
